# Graft Immune Cell Composition Associates with Clinical Outcome of Allogeneic Hematopoietic Stem Cell Transplantation in Patients with AML

**DOI:** 10.3389/fimmu.2016.00523

**Published:** 2016-11-21

**Authors:** Ulla Impola, Antti Larjo, Urpu Salmenniemi, Mervi Putkonen, Maija Itälä-Remes, Jukka Partanen

**Affiliations:** ^1^Finnish Red Cross Blood Service, Research and Development, Helsinki, Finland; ^2^Turku University Central Hospital, Turku, Finland

**Keywords:** GVHD, AML, transplantation, immune cells

## Abstract

Complications of allogeneic hematopoietic stem cell transplantation (HSCT) have been attributed to immune cells transferred into the patient with the graft. However, a detailed immune cell composition of the graft is usually not evaluated. In the present study, we determined the level of variation in the composition of immune cells between clinical HSCT grafts and whether this variation is associated with clinical outcome. Sizes of major immune cell populations in 50 clinical grafts from a single HSCT Centre were analyzed using flow cytometry. A statistical comparison between cell levels and clinical outcomes of HSCT was performed. Overall survival, acute graft-versus-host disease (aGVHD), chronic graft-versus-host disease (cGVHD), and relapse were used as the primary endpoints. Individual HSCT grafts showed considerable variation in their numbers of immune cell populations, including CD123^+^ dendritic cells and CD34^+^ cells, which may play a role in GVHD. Acute myeloid leukemia (AML) patients who developed aGVHD were transplanted with higher levels of effector CD3^+^ T, CD19^+^ B, and CD123^+^ dendritic cells than AML patients without aGVHD, whereas grafts with a high CD34^+^ content protected against aGVHD. AML patients with cGVHD had received grafts with a lower level of monocytes and a higher level of CD34^+^ cells than those without cGVHD. There is considerable variation in the levels of immune cell populations between HSCT grafts, and this variation is associated with outcomes of HSCT in AML patients. A detailed analysis of the immune cell content of the graft can be used in risk assessment of HSCT.

## Introduction

Allogeneic hematopoietic stem cell transplantation (HSCT) is an established and often the only curative treatment for many malignant hematological diseases. Allogeneic HSCT grafts are harvested from bone marrow (BM) or from peripheral blood (PB) after HSC mobilization and transplanted to the patient in order to reconstitute hematopoiesis and the immune system with the donor’s cells and to immunologically kill the recipient’s residual cells. Graft-versus-host disease (GVHD) is a severe complication of allogenic HSCT in which the donor’s immune cells recognize the patient’s tissues as foreign and destroy epithelial and in some cases also endothelial cells. The acute form of GVHD (aGVHD) normally occurs within the first 100 days after engraftment, whereas symptoms of chronic GVHD (cGVHD) appear later (1, 2). The pathogenesis of GVHD involves complex interactions of cytokines and immune cells; however, the exact mechanism is not completely understood (2–5). The donor’s alloreactive effector T cells and the host’s antigen-presenting cells (APCs), such as dendritic cells, have been shown to have an essential role in the initiation of GVHD. Depletion of effector T cells from the HSCT graft decreases the risk of GVHD but at the same time increases the relapse rate, obviously due to insufficient graft-versus-leukemia (GVL) effect (2, 4–6).

Studies performed mostly on animal models have demonstrated the role of donor’s immune cells in more detail (5, 7–10). Effector T cells, in particular alloreactive CD8^+^ T cells, of the graft are known to have a crucial, direct role in the pathogenesis of GVHD (5, 11). As well as the dose of regulatory T cells (12–14), the balance between the Th1 and Th2 subsets of T cells is thought to be critical in prevention and control of the intensity of GVHD (5, 15). Matte et al. showed that the donor’s APCs are required for GVHD but not for the GVL effect (16). Furthermore, a high number of B cells in the graft have been reported to associate with the aGVHD risk. B cells most likely present antigens to T cells and secrete activating cytokines (17, 18), whereas monocytes and invariant NKT cells are thought to have an anti-GVHD effect (8, 9). The dose per patient kilogram of CD34^+^ hematopoietic progenitor cells is applied in clinical practice as a high dose of CD34^+^ cells is often reported to lead to a better hematopoietic recovery and transplantation outcome, although also conflicting effects are reported and there are contradictory suggestions as an optimal CD34^+^ dose (19–22). At least in some donor-recipient combinations, natural killer (NK) cells are effective in killing remaining malignant cells and hence decrease the risk of relapse (23–25).

The immune cell composition of the graft is typically not characterized in clinical practice, except for the numbers of CD34^+^ and CD3^+^ cells. Obviously, there is a high level of variation in the cellular composition of the grafts, depending on the donor’s background and stem cell collection methods and source (11), although only a few systematic, modern studies have been published (26). To address this question, we analyzed variation in the levels of immune cell populations in 50 clinical HSCT grafts and its impact on the outcome of allogenic HSCT.

## Materials and Methods

### Patients

Altogether, 73 allogeneic HSCTs were performed for adult patients in the Turku Central Hospital Hematology Ward and Stem Cell Transplantation Unit during the study period between February 2014 and February 2015. For the present study, we were able to get fresh samples of sufficient size from 50 grafts.

The demographic data of the 50 patients and the 50 HSCTs carried out on them are shown in Table 1. It is of note that one patient, in fact, received two HSCTs, only the first of which was included in the present study. A 46/50 (94%) patients received a 10–12/12 HLA-matched transplants. Nine (18%) patients received a graft from a related donor and 39 patients (78%) from a matched unrelated donor. Two patients (4%) were transplanted with a graft from a haploidentical (6/12 matched) family donor.

**Table 1:** **The demographic data of the 50 patients and the 50 HSCTs included in the study**.

Disease	Frequency	Gender F/M	Median age (*x*–*y*)	Graft source BM/PB	Conditioning RIC/MAC (seq.)	IS cni/mtor	aGVHD/grades 3–4	cGVHD yes/no (ne)	Relapse	CMV	Alive 9/2015
ALL	6	5/1	32 (19–66)	1/5	1/5	4/2	3/0	2/3 (1)	1	3	5
AML	23	10/13	57 (29–68)	5/18	10/8 (5)	6/16^a^	13/6	7/12 (4)	4	12	15
CLL	3	1/2	61 (58–61)	0/3	2/1	1/2	3/2	2/1	0	1	3
MDS	3	1/2	60 (58–63)	1/2	2/1	1/2	1/1	2/1	0	2	2
MF	1	1/0	51	0/1	1/0	0/1	1/1	(1)	1	1	0
MM	7	4/3	55 (43–62)	0/7	3/4	2/5	5/4	5/2	0	5	5
MPN	1	0/1	43	0/1	0/1	0/1	0	(1)	1	1	0
SAA	3	3/0	55 (22–63)	3	4/0	3/1	3/0	1/2	1	1	2
MbH	1	0/1	43	0/1	0/1	0/1	0	0/1	0	0	1
NHL	1	0/1	24	0/1	1/0	0/1	0	1/0	1	0	0
T-LBL	1	0/1	64	0/1	0/1	0/1	1/1	1/0	1	0	0
Total	50	25/25	55 (19–68)	10/40	24/21 (5)	17/33	30/15	21/22 (7)	9	26	33

*Patient hematological malignancies varied a lot. Primary diseases were acute myelogenous (AML) and lymphoblastic leukemia (ALL), chronic lymphoblastic (CML) and myelomonocytic (MM) leukemia, myelodysblastic syndrome (MDS), myelofibrosis (MF), myeloproliferative neoplasms (MPN), severe aplastic anemia (SAA), Hodgkin (MbH) and non-Hodgkin lymphomas (NHL), and T-cell lymphoblastic lymphoma (T-LBL)*.

*ne, not evaluable*.

*^a^One patient received steroid treatment*.

Informed consent was obtained from all patients, and the study protocol was approved by the local Ethical Review Board of the Turku University Hospital, Turku, Finland.

### Clinical Protocols, Data, and Endpoints

The patients were treated with the standard clinical protocols. They were pre-conditioned by using myeloablative chemotherapy (MAC), reduced-intensity conditioning (RIC), or sequential use of intensive chemotherapy (SEQ). The MAC regimen consisted of fludarabine 30 mg/m^2^/day for 5 days and busulfan 3.2 mg/kg/day intravenously for 3–4 days, or, for patients with ALL, cyclophosphamide 60 mg/kg/day for 2 days, followed by fractionated total body irradiation 12 Gy. The RIC regimen consisted of fludarabine 30 mg/m^2^/day for 5 days and busulfan 3.2 mg/kg/day intravenously for 2 days. The SEQ conditioning included fludarabine-high dose sytarabine-idarubicine induction, followed by conditioning with cyclophosphamide 60 mg/kg/day for 2 days and total body irradiation 4 Gy. Rabbit antithymocyte globulin at a dose of 2.5 mg/kg/day for 2 days together with tacrolimus or everolimus and a short course of methotrexate were used for GVHD prophylaxis.

Clinical data were collected routinely and included in the study during the surveillance period until the end of September 2015. The median follow-up period was 12 months (range 7–19 months). GVHD was defined as either acute (Seattle-Glucksberg criteria) (27) or chronic according to the standard criteria and on the basis of the 100-day onset limit, unless the patient had characteristic symptoms of acute GVHD after day 100 (late onset aGVHD).

Acute GVHD was graded from none or mild (grades 0–1) to severe/life-threatening GVHD (grades 2–4) according to Przepiorka et al. (27). Chronic GVHD was classified for statistical analysis as yes or no.

Patient chimerism was tested monthly from whole blood and from the blood T-cell fraction during the post-transplant period and from the BM CD34^+^ precursor cell fraction at 2- to 3-month intervals by highly sensitive Abbott AlleleSEQR chimerism assay. Serum cytomegalovirus was monitored weekly during the first 6 months by qPCR.

### HSCT Graft Samples

An aliquot of 3–4 ml was taken from allogeneic HSCT grafts before infusion. The fresh sample was transported to the FRC Blood Service and analyzed within 12 h. Briefly, sample was diluted 1:1 in PBS-EDTA (Invitrogen) supplemented with 2% (V/V) fetal bovine serum (Gibco), overlaid onto Ficoll-Paque PLUS (GE Healthcare) density gradient medium, and centrifuged in a SepMateTM-15 tube (StemCell Technologies) according to the manufacturer’s instructions. The mononuclear cell (MNCs) fraction, buffy coat, was collected and analyzed. Cell viability and the total number of cells were calculated with NucleoCounter before further analysis.

### Flow Cytometric Analysis

Relative proportions of each cell populations in the aliquot of graft sample were analyzed using flow cytometry [FACSAria IIu, Becton Dickinson (BD)] independently at the Finnish Red Cross Blood Service as part of the present study. Cell populations of different main immune cell populations and CD34^+^ hematopoietic progenitor cells (hereafter “CD34^+^ relat”) were identified using combinations of fluorochrome-labeled antibodies (see Table 2). Percent amount of each cell population was calculated as a proportion out of CD45^+^ cells. Average proportions of lymphocyte and monocyte cell populations were estimated and gated based on their size and granularity.

**Table 2:** **Immune cell subpopulations and their surface markers**.

Immune cell subsets	Definition
Hematopoietic cells	CD45^+^
B cells	CD45^+^, CD19^+^
Myeloid dendritic cells	CD45^+^, CD11c^+^, CD19^−^, CD123^−^
Plasmacytoid dendritic cells	CD45^+^, CD11c^−^, CD19^−^, CD123^+^
CD34^+^ progenitor cells	CD45^+^, CD34^+^
CD3^+^ T cells	CD45^+^, CD3^+^
CD8^+^ T cells	CD45^+^, CD3^+^, CD4^−^, CD8^+^
CD4^+^ T cells	CD45^+^, CD3^+^, CD4^+^, CD8^−^
CD25^+^ T cells	CD45^+^, CD3^+^, CD4^+^, CD8^−^, CD25^+^
CD3^−^ cells	CD45^+^, CD3^−^
Natural killer cells	CD45^+^, CD3^−^, CD16^+^, CD56^+^
Lymphocytes	FSC, SSC, CD45^+^, CD3^+^
Monocytes	FSC, SSC, CD45^+^

*Immune cell subpopulations were identified using combinations of fluorochrome-labeled antibodies. Cell populations were gated based on their combination of cell surface markers, cell size, and granularity*.

Antibody-fluorochrome combinations included CD45-PerCp-Cyanine 5.5, CD3-FITC, CD4-APC-eFluor780, CD11c-APC, CD16-PE, CD19-FITC, CD25-BV421, CD34-PE, CD56-APC (all from eBiosciences), and CD123-BV421 and CD25-BV421 (Becton Dickinson). Conjugated isotype controls (eBiosciences) and unlabeled cells were used as negative controls. BD Compensation beads were used for signal compensations. Instrument settings and performance were checked using BD’s CST Protocol. The FACSDiva™ Flow Cytometry Software Version 5.02 and FlowJO 7.0 were used for data analysis.

The total number of CD34^+^ cells in the entire volume of the collected graft (hereafter “CD34^+^ tot”) was measured in the stem cell collecting centers during harvesting of the graft. In our sample, cohort the median total, number of CD34^+^ cells was 1.48 × 10^8^ cells/l in BM- and 5.82 × 10^8^ cells/l in PB-derived grafts. The total number of CD34^+^ cells × 10^8^/l was used for the determination of clinical dose (CD34^+^ dose, number of CD34^+^ cells per recipient weight).

### Colony Forming Unit Assay

The Colony Forming Unit (CFU) assay estimates the number and potency of hematopoietic progenitors. The assay was performed according to the manufacturer’s instructions (MethoCult™ Media, StemCell Technologies). Cell colonies were counted on days 13 and 18, and triplicates were used in calculations.

### Statistical Analysis

Statistical tests on associations between levels of cell populations in grafts and clinical endpoints were mainly carried out using Wilcoxon rank-sum test and Fisher’s exact test, and other tests as described in the text. As multivariate analysis was not meaningful due to low numbers of cases in some groups, we instead used random forest classifiers to assess how well the clinical endpoints could be predicted using the measured cell populations while also including other possibly influencing factors. R and caret package (28–30) were used for statistical analyses. All cell populations, as well as HLA match, graft type, immunosuppressive medication, pre-conditioning, and donor type, were used as potential predictors, and the accuracies were estimated using fourfold cross-validation repeated 100 times. Within each clinical endpoint, we ranked (from 0 to 100) the importance of the parameter as a predictor. These rankings were used along with the nominal *p*-values to estimate the significance of the findings. It is of note that the results are based on a limited set of data points and the classifiers have not been tested with real held-out dataset, so the reported performances (Table S1 in Supplementary Material) may be over-optimistic.

## Results

### Clinical Outcomes

The demographic data of the patients are summarized in Table 1. During the study period, 17 of the 50 patients died (overall survival 66%, transplantation related mortality 24%). Patients who received stem cell graft from an HLA-matched sibling donor had a better overall survival than those who received an unrelated HSCT (*p* = 0.039). No other statistically significant association was found between clinical endpoints and the donor type. The HLA match showed no statistically significant association with clinical endpoints (data not shown).

Acute GVHD was diagnosed in 30 of the 50 patients (60%), 15 of whom had severe grades 3–4 aGVHD while 15 had grades 1–2 aGVHD. Chronic GVHD was classified as yes/no, and even very mild symptoms were included to the positive group. This might explain rather high incidence of cGVHD among our patients as twenty-one (42%) had cGVHD. Nine of the 50 patients (18%) had a relapse during the surveillance period. Serum cytomegalovirus was found in 26 of the 50 patients (52%).

### Variation between BM and PB Grafts

**Figure 1:**
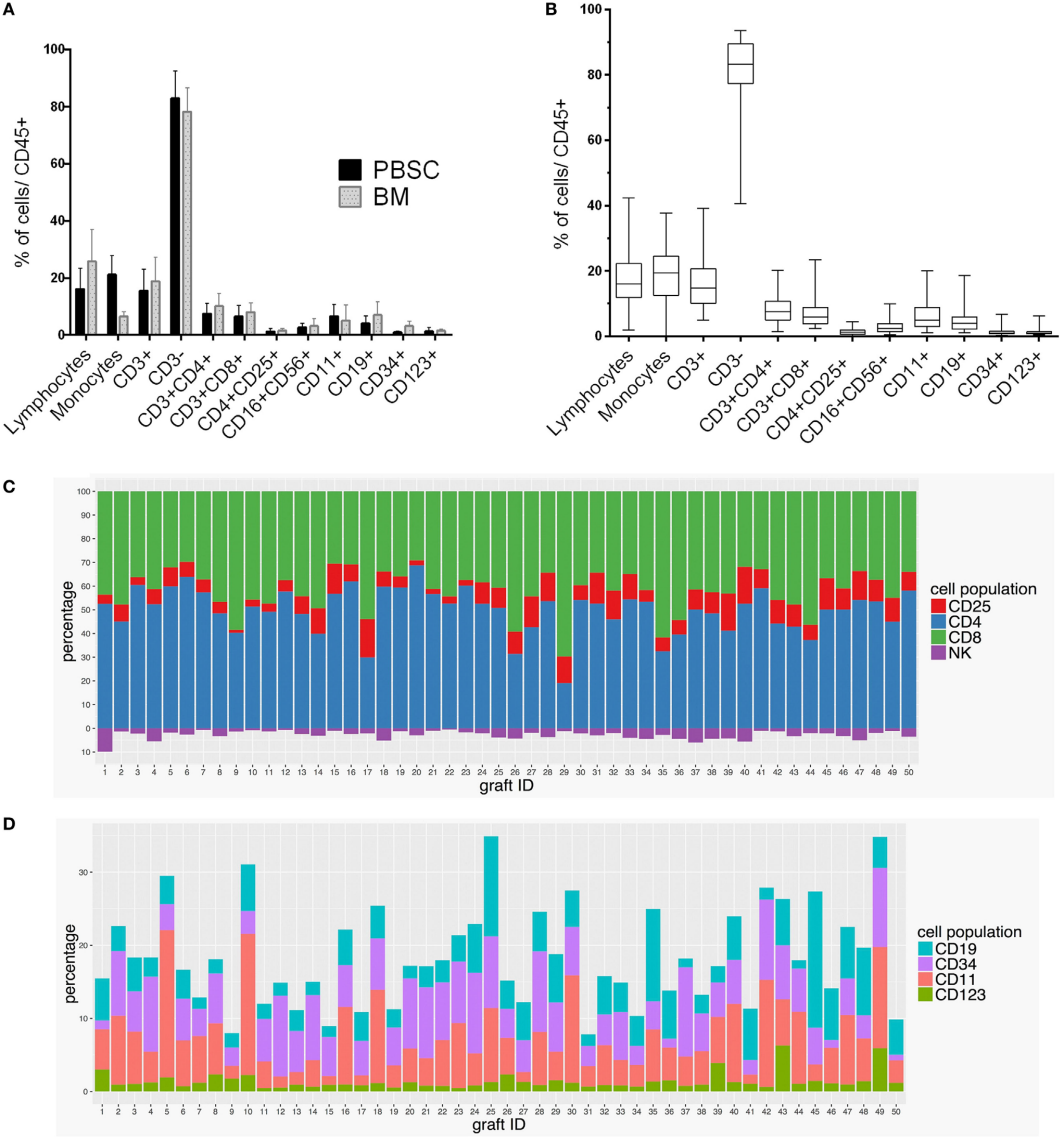
**Variation of immune cell subpopulations in HSCT grafts**. The composition of immune cell populations varied **(A)** among BM and PB grafts and **(B)** in individual 50 clinical HSCT grafts as measured by the percentages of the cell populations in graft samples. **(C)** Levels of CD3^+^, CD4^+^, CD8^+^, CD4^+^ CD25^+^, CD16^+^ CD56^+^ cell subpopulations and **(D)** CD19^+^, CD34^+^, CD11c^+^, and CD123^+^ cell subpopulations in each HSCT graft (*N* = 50) were estimated as percentages out of CD45^+^ cells. Lymphocytes and monocytes were identified based on their size and granularity.

The grafts originated from BM in 10 HSCTs (20%) and from PB in 40 HSCTs (80%). We compared the differences in the proportional shares, i.e., not absolute numbers, of immune cell populations between these two types of grafts (Figure 1A). BM-derived grafts contained higher levels of lymphocytes (*p* = 0.008), CD34^+^ cells (*p* = 4.3 × 10^−6^), and CD19^+^ B cells (*p* = 0.009) than the PB-derived grafts. PB-derived grafts had a significantly higher level of monocytes (*p* = 2.4 × 10^−10^) than the BM grafts. The graft type as such was not found to be associated with the clinical endpoints.

### Variation between Individual Grafts

The levels of immune cell subpopulations varied between individual stem cell grafts (Figures 1B–D). Figure 1B shows the variation of all cell populations, and Figure 1C shows the variation in the effector subsets of CD3^+^ cells and in NK cells. Figure 1D shows the change in the levels of B cells, CD34^+^ cells, and two types of dendritic cells (CD11^+^ and CD123^+^ cells). The levels of many cell populations known to be relevant to the outcome of HSCT showed variation between the grafts. The highest coefficients of variation (variability in relation to the mean of the measurement) were seen for the levels of CD34^+^ progenitor cells, CD123^+^ plasmacytoid and CD11c^+^ myeloid dendritic cells, and CD19^+^ B cells. On the other hand, CD4^+^ and CD8^+^ T-cell populations had low coefficients of variation (data not shown).

### Association of Variation in the Graft Cell Populations with Clinical Endpoints

Association analyses between the clinical endpoints and levels of immune cells in the grafts were performed. As multivariant analysis was not meaningful due to low numbers of cases in some groups, we instead used random forest classifiers to assess how well the clinical endpoints could be predicted using the measured cell populations while also including other possibly influencing factors. Hence, classifiers were built to find how important each cell population is for predicting clinical outcomes. The best classification accuracies were obtained in Acute myeloid leukemia (AML) cases for cGVHD (AUC 0.86), relapse (AUC 0.81), and aGVHD (AUC 0.78).

When all diagnoses were included, none of the classifiers achieved a very high performance (maximum AUC 0.73), possibly indicating high heterogeneity in the diseases. All results are summarized in Table S1 in Supplementary Material.

**Figure 2:**
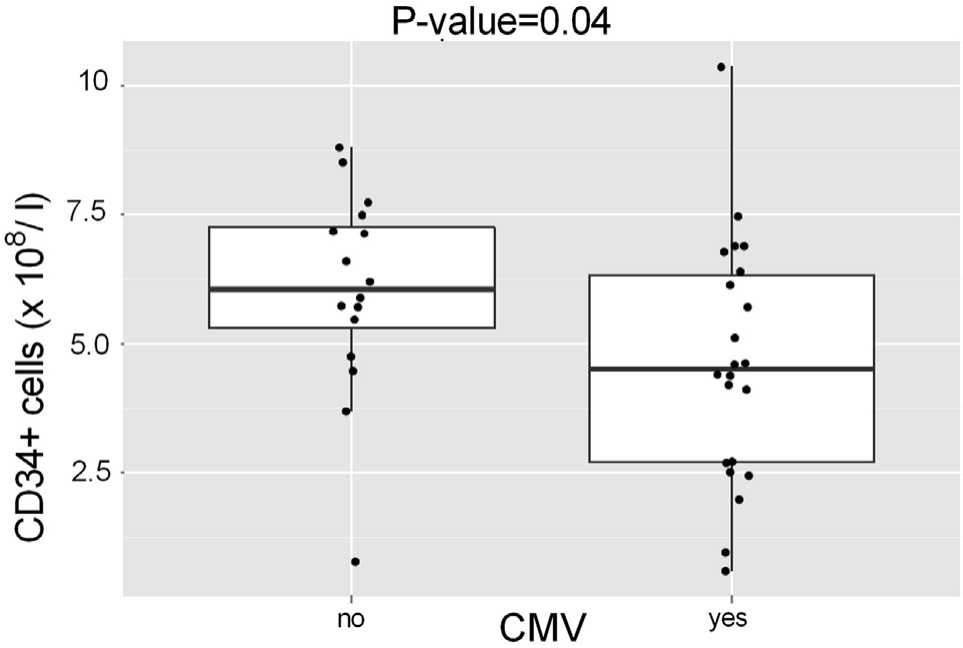
**Association of CD34^+^ cell numbers in the HSCT grafts with serum cytomegalovirus**. Patients (*N* = 26) with serum cytomegalovirus had received HSCT grafts that contained lower total number of CD34^+^ cells (CD34^+^ tot, median 5.3 × 10^8^/l) than those with no detected cytomegalovirus (*N* = 24) *p* = 0.04.

When all HSCTs were included, a low “CD34^+^ tot” level in the graft was associated with the cytomegalovirus positivity (Figure 2; *p* = 0.04). In addition, an association was observed between a low level of “CD34^+^ relat” and the occurrence of cGVHD (*p* = 0.022) among the patients (*N* = 10) who received a BM graft. A low level of CD25^+^ CD4^+^ T cells, a population containing regulatory T cells, was associated with the occurrence of relapse (*p* = 0.041) and rejection (*p* = 0.032) (data not shown).

**Figure 3:**
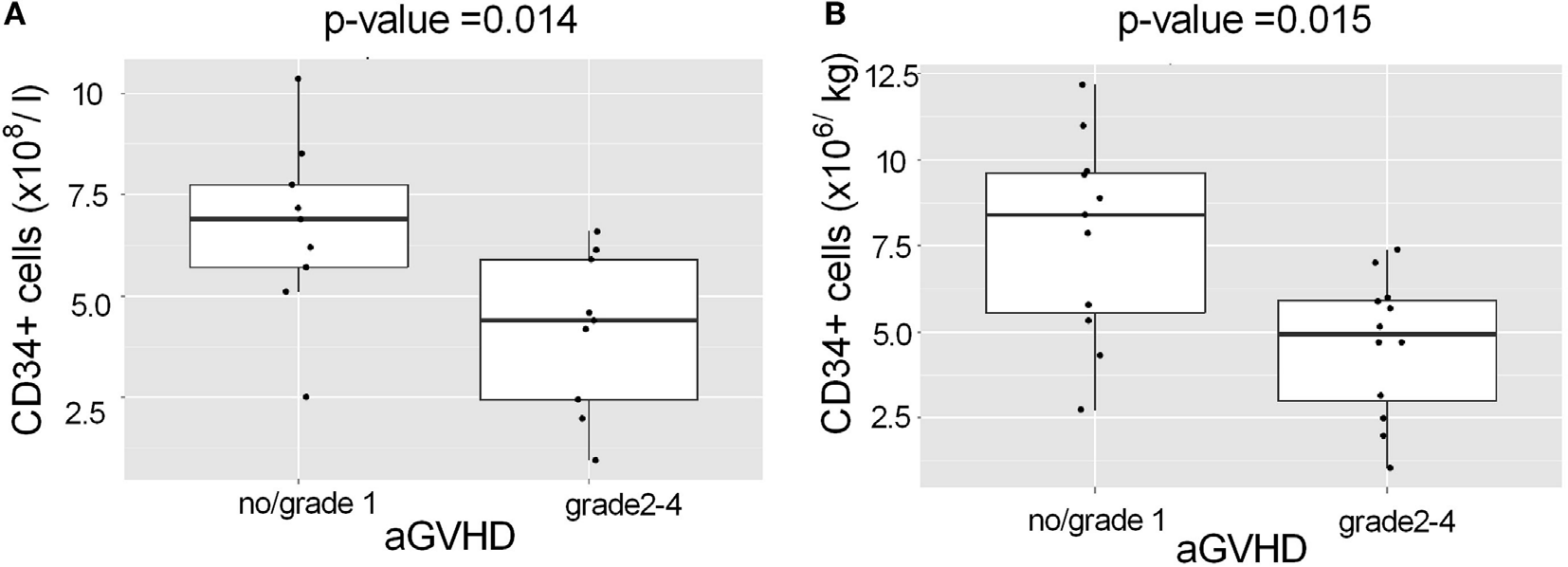
**Association of CD34^+^ cell levels in the HSCT grafts with aGVHD among AML patients**. AML patients with grades 2–4 aGVHD had received HSCT grafts **(A)**, which contained a lower total number of CD34^+^ cells (*p* = 0.014) and **(B)** a lower dose of CD34^+^ cells/kg (*p* = 0.015) than AML patients with grade 1 or no aGVHD. The median dose of CD34^+^ cells/kg of recipient was 5.7 × 10^6^/kg.

To reduce heterogeneity of the patient group, we analyzed the largest single patient group, AML, separately (*N* = 23 of the 50 patients). A low “CD34^+^ tot” level in the graft (Figure 3A; *p* = 0.014) and a low CD34^+^ dose (cells/kg; Figure 3B; *p* = 0.015) were both associated with the occurrence of aGVHD in the AML patients. The association remained statistically significant regardless of conditioning (data not shown).

**Figure 4:**
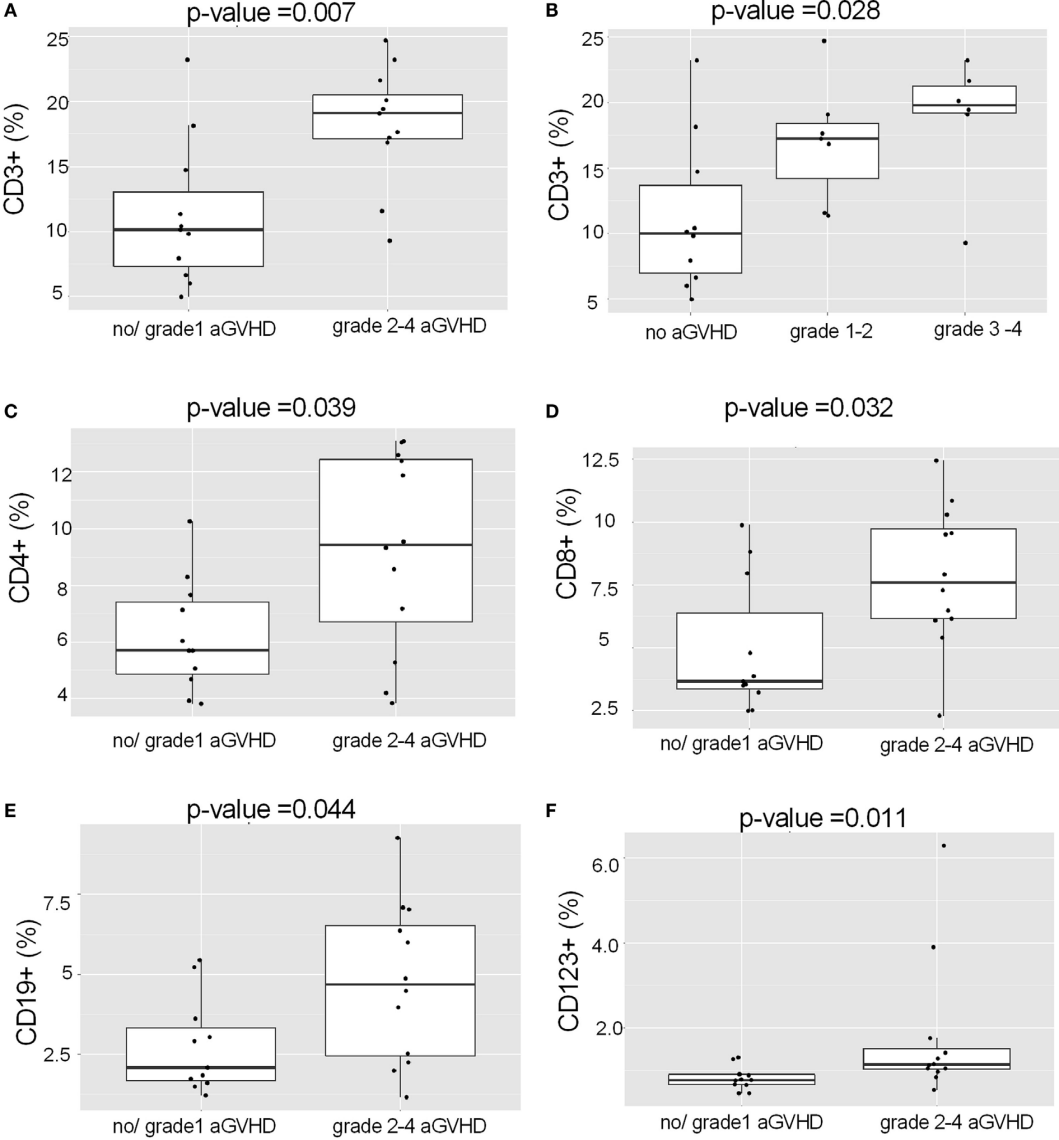
**Association of the graft proportions of CD3^+^ lymphocytes, CD19^+^ and CD123^+^ cells with acute GVHD in AML patients**. **(A)** AML patients with grades 2–4 aGVHD had received grafts with higher levels of CD3^+^ cells than patients with grade 1 or no aGVHD (*p* = 0.007). **(B)** Patients with AML showed a trend (*p* = 0.028, Kruskal–Wallis test) toward more severe aGVHD along with the increasing levels of CD3^+^ cells in the graft. AML patients with grades 2–4 aGVHD had received HSCT grafts with higher levels of **(C)** CD4^+^, **(D)** CD8^+^, **(E)** CD19^+^, and **(F)** CD123^+^ cells as compared to those with grade 1 or no aGvHD. All proportions are calculated as %/CD45^+^ cells.

Acute myeloid leukemia patients with grades 2–4 aGVHD had received grafts with higher levels of CD3^+^ T lymphocytes than those without aGVHD (Figure 4A; *p* = 0.007). In fact, there was a clear trend between the levels of CD3^+^ cells and the severity of aGVHD (Figure 4B; *p* = 0.028; Kruskal–Wallis test). Occurrence of grades 2–4 aGVHD in AML patients was associated with high levels of many effector immune cells in the graft: CD4^+^ cells (Figure 4C; *p* = 0.039), CD8^+^ cells (Figure 4D; *p* = 0.032), CD19^+^ cells (Figure 4E; *p* = 0.044), and CD123^+^ (Figure 4F; *p* = 0.011). Of these, particularly CD8^+^ and CD123^+^ ranked high as predictors, attesting to the prominence of their role.

**Figure 5:**
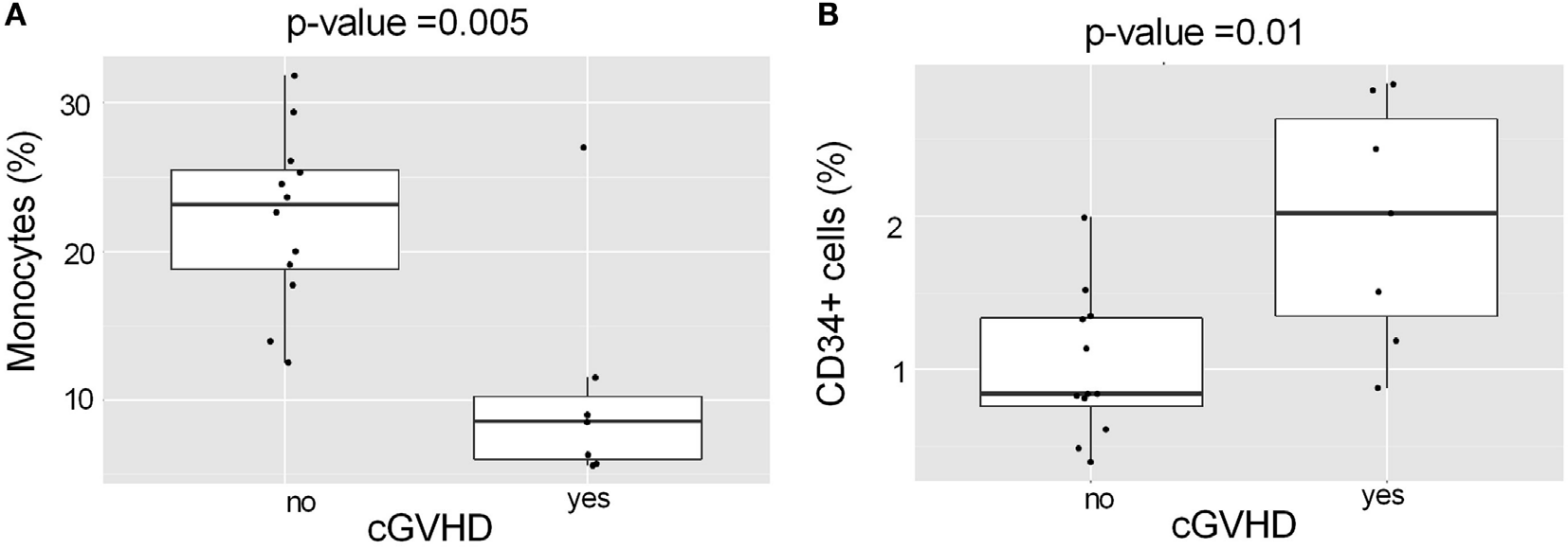
**Association of monocyte and CD34^+^ cell levels in the HSCT grafts with chronic GVHD in AML patients**. **(A)** AML patients with cGVHD had received HSCT grafts with lower levels of monocytes than AML patients without cGvHD (*p* = 0.005). **(B)** AML patients with cGVHD had received HSCT grafts with higher levels of CD34^+^ cells than those without cGVHD (*p* = 0.01).

Acute myeloid leukemia patients with cGVHD had received HSCT grafts containing lower levels of monocytes (Figure 5A; *p* = 0.005) and higher levels of “CD34^+^relat” (Figure 5B; *p* = 0.01) than those without cGVHD. Monocytes were ranked as the most important predictor for cGVHD, indicating that they play a major role.

## Discussion

Graft-versus-host disease remains a major challenge in allogeneic HSCT. The mechanism of GVHD is complex and depends on many factors, particularly on the interactions of various immune cell populations and on the donor’s T cells that are transferred along with the allograft and which attack host tissues (5). Hence, it is plausible that the composition of immune cells in the clinical HSCT graft also influence the outcome. However, current clinical HSCT protocols usually rely on counting from the graft only the numbers of nucleated cells and CD34^+^ hematopoietic stem cells, with estimates of CD3^+^ T lymphocytes sometimes included. No detailed analysis of cellular content of the graft is performed. To address the role of immune cell composition of the graft, in more detail, we studied systematically 50 clinical HSCT grafts for their immune cell composition and its influence on the clinical outcome of HSCT.

First, we found that there was considerable variation between individual grafts in regard to levels of many cell populations that, based on models of GVHD pathogenesis (3, 5, 10, 31), can be assumed to influence the clinical outcome of HSCT, such as the severity of GVHD or incidence of relapse. Second, we present evidence that this variation in levels of certain cell populations, in particular levels of effector immune cells, is significantly associated with the grade and type of GVHD. The effect was clearer in a subgroup of patients, those with AML, suggesting that the underlying disease and its treatment should be taken into account.

There are only a few recent publications (11, 26, 32, 33) describing systematically variation in the levels of immune cell populations in clinical HSCT grafts. Many studies have concentrated on the effects of stem cell mobilizing regimens on immune cell levels. In these studies, differences were reported in the numbers of T lymphocytes and their Th1/Th2 balance (34) and NK cells and dendritic cells (11, 35, 36). Recent studies (9, 37) found that mobilization induced particular subpopulations of monocytes that may regulate GVHD. In our present study, we observed the highest levels of variation in CD34^+^ hematopoietic cells, CD123^+^ plasmacytoid and CD11c^+^ myeloid dendritic cells, CD19^+^ B cells, and CD4^+^CD25^+^ T cells; all these cell populations can be assumed to play a role in GVHD, GVL, or relapse. As an example, the levels of CD11^+^ positive dendritic cells differed up to 10-fold between individual grafts. As relatively small numbers of dendritic cells can activate the immune response by presenting peptide antigens to naïve T cells, the differences observed here could be immunologically relevant (38, 39).

Only a few studies have directly addressed the origin of variation in immune cell numbers. It is obviously possible that genetic factors, such as HLA genes or interleukin-2 receptor A gene, determine the size of at least some immune and blood cell populations as reported by e.g., Orrú et al. (40). Orrú et al. concluded that heritability accounted for up to 87% of the variation in immune cell levels (40). However, Brodin et al. reported (41) based on a twin study that in fact, non-heritable factors, such as immunological history and vaccinations, were the major drivers of variation in the human immune system. How much these studies can be extended to clinical HSCT grafts is unclear.

The origin of the HSCT graft, BM versus PB is an obvious source of variation in cell content. PB has become widely used as a stem cell source, almost replacing the use of BM in allogeneic HSCT in some centers (35). In the present study, 20% of the grafts originated from BM and the rest were harvested from mobilized PB. The PB-derived grafts had a significantly higher level of monocytes than BM-derived grafts; this finding is in line with those by Korbling and Freireich (35) and Ottinger et al. (42). The BM-derived grafts of the present study contained higher levels of lymphocytes, CD19^+^ B cells, and CD34^+^ cells. The higher level of CD34^+^ cells in BM has been established earlier (43), whereas B cells have been reported to be more frequent in PB than in BM (43, 44).

The variation in the cell levels between the grafts was found to influence the clinical outcome of HSCT. It is of interest that the highest level of variation in the present study was seen in the number of CD34^+^ hematopoietic cells, the very same population that appears to be one of the major determinants of the outcome of HSCT. The median total number of CD34^+^ cells in BM grafts was in the present study about fourfold lower than that in PB-derived grafts. However, the relative proportion of CD34^+^ cells in BM samples was clearly higher compared to PB grafts. We found that high levels of CD34^+^ cells in the graft protected from severe aGVHD but predisposed to cGVHD in AML patients and protected from serum cytomegalovirus in all patients. A high dose of CD34^+^ cells has been reported to lead to a better hematopoietic recovery, however not always to a better transplantation outcome (19, 20, 22) Conflicting data is presented also in other studies (21, 45). Our finding that a high CD34^+^ cell content in the graft predisposed to cGVHD may be related to CD34^+^-derived dendritic cells that have been reported to be able to augment antigen presentation to donor T cells and induce cGVHD (46). It is of note that according to a recent study by Martin et al. (47), estimates of the total number of nucleated cells, especially CD34 negative cells, might in fact be better predictors of HSCT outcome than the CD34^+^ cell dose at least in HSCT performed using RIC and PB.

Another major finding in the present study was the association of high levels of many effector cell populations, such as CD3^+^, CD4^+^, and CD8^+^ T cells or CD19^+^ B cells, in the HSCT grafts with the more severe aGVHD in AML patients. In addition, AML patients with severe aGVHD had received higher levels of plasmacytoid CD123^+^ dendritic cells (pDCs) in the grafts than those without or with grade 1 aGVHD. The association of high levels of effector cell populations with more severe aGVHD is not surprising as it is assumed that many effector cell populations of donor-origin are involved in the pathogenesis of aGVHD (1, 5, 15). There is an abundance of evidence on the role of T cells as initiators of aGVHD. Also, as mediators of antitumor immunity, CD8^+^ cells, when present in high doses, are known to be associated with a lower risk for relapse (48), but we could not verify this in the present study. High regulatory T cell doses are known to control GVHD while still allowing active GVL effect (12, 13, 49, 50). We found some evidence indicating that low doses of CD4^+^ CD25^+^ T cells, a population including regulatory T cells, were associated with higher risks for relapse and rejection.

The possible role of pDCs in inducing aGVHD is interesting, but so far these cells have not been studied extensively in this context (26, 51). pDCs constitute a multifunctional cell type involved e.g., in production of type-I interferons against virus infections, induction of Th17 cell response, and presentation of peptide antigens by MHC class II to T cells (52). High levels of pDCs in the graft can lead to immune activation as pDCs may more readily detect, *via* their toll-like receptors, the tissue damage caused by pre-transplantation conditioning. Then, they may become activated and act as APC. However, experimental data for this is still scarce (51, 53). Peric et al. recently reported that high levels of pDCs post-HSCT predicted good clinical outcome with less severe GVHD and better overall survival (53). Waller and coworkers (26) found that survival was better in HSCTs with high pDCs. More research on the role of pDCs in GVHD is clearly warranted.

Clinical presentation of cGVHD resembles fibrotic autoimmune disorders and involves Th2 and B cells (54), cytokines secreted by Th1 cells (55), Th17 cells, and autoantibodies (54). Also, a low number of active regulatory T cells (56) have previously been associated with cGVHD. The levels of regulatory T cells or B cells in the graft were not associated with cGVHD in the present study. However, we found that low levels of CD34^+^ cells and monocytes in the graft were associated with cGVHD in AML patients. The CD34^+^ and monocyte populations can be regarded as a source of dendritic cells (57), which can efficiently present antigens to donor T cells and may, therefore, be involved in the induction of cGVHD. Our finding that various cell populations in the grafts were found to be associated with the development of aGVHD, as opposed to cGVHD, supports distinct immunological background and pathogenesis between the two types of GVHD.

The present study demonstrates a considerable variation of the cellular content in the HSCT graft which might affect patient outcome depending on their diagnosis. In addition to the numbers of CD34^+^ and CD3^+^ cells, a more detailed profiling of graft immune cells and their proportions might provide beneficial knowledge of cell populations that play a role in the pathogenesis of GVHD. This could be applied in risk assessments in HSCT and support the development of more personalized transplantation protocols.

## Author Contributions

UI, MI-R, and JP designed the research; MP, US, and MI-R treated the patients and collected the samples and clinical data; UI performed laboratory analyses with flow cytometry; AL did statistical analysis; UI, AL, JP, and MI-R interpreted the results; and UI, AL, JP, and MI-R wrote the manuscript.

## Conflict of Interest Statement

The authors declare that the research was conducted in the absence of any commercial or financial relationships that could be construed as a potential conflict of interest.
